# Seed plant diversity of different forest types in Liangshui National Natural Reserve

**DOI:** 10.3897/BDJ.5.e22167

**Published:** 2017-12-29

**Authors:** Hongfeng Wang, Xueyun Dong

**Affiliations:** 1 School of Forestry, Northeast Forestry University, Harbin, China; 2 School of Food Engineering Harbin University, Harbin, China

**Keywords:** China, diversity, quadrat, Liangshui Natural Reserve, conservation, species inventory

## Abstract

**Background:**

Thirty years ago, there was a monograph of vegetation and plant diversity in the region prepared by the Department of Forestry at the Northeast Forestry University (unpublished), but the variety of plants in the region has changed significantly over the past 30 years. In future years, the authors hope to publish a new monograph and this research is to prepare for this work. This study aimed at reporting the characteristics of plant diversity in five different forest types in Liangshui National Natural Reserve, China, each with three 25 × 25 m tree quadrats, twelve 5 × 5 m wide shrub quadrats and twelve 1 × 1 m wide herbaceous quadrats. Censuses of each forest type were conducted in 2016.

**New information:**

The five main forest types presented differences in structure, diversity and species richness.

## Introduction

The Korean pine forest of Liangshui National Natural Reserve is the most important primitive broad-leaved Korean pine forest in China and it is also one of the modern Korean pine distribution centres (Liu 2015, Zhang et al. 2000). The primitive broad-leaved Korean pine forest area is less than 40000 hm^2^ in China (Liu et al. 2014), while there are 4100 hm^2^ in Liangshui National Natural Reserve, accounting for 11% of the country (Liu et al. 2014). Liangshui National Natural Reserve is rich in biodiversity, preserves several species of the Tertiary plant communities and has an irreplaceable role in maintaining global diversity (Chen et al. 2012, Liu et al. 1993, Ma et al. 2007, Wang 1981, Rong et al. 2009). Korean pine forest has existed on earth for at least 30 million years, but the existing broad-leaved Korean pine forests of the Reserve formed about 3000 years ago. The Korean pine forest in this area is young, the age of the oldest individual is no more than 500 years, with a 1.5 m breast diameter. The tree height here is 15-40 m and seeds produced per plant are about 30 kg (Liu 2015). Although the Liangshui National Nature Reserve is very important, research for the different forests is not comprehensive and the data of plant diversity are still insufficient. In this research, the plant diversity of five different forest types in this area were studied, which provided the basic data for the study of biodiversity in this area.

## Project description

### Title

Construction of Plant checklist Database in Heilongjiang Province

### Personnel

Wang Hongfeng

### Study area description

Liangshui National Natural Reserve is located in the southeastern of the Xiaoxing'anling Mountains in Heilongjiang Province, China (47°7'39"-47°14'22" N, 128°48'30"-128°55'50" E). It is made up of hills, the terrain is high in south and low in north. The average elevation is about 400 m, the highest elevation is 707.3 m, the lowest is 280 m, mountain slope is generally 10-15 degrees (The Compilation Group Physical Geography of China 1986). This area belongs to the temperate continental monsoon climate. The extreme minimum temperature is -43.9 °C and the extreme maximum temperature is 38.7 °C. The annual average temperature is about -0.3 °C, positive temperature accumulated is 2200-2600°C. The average annual precipitation is 676 mm, June-August precipitation accounted for more than 60% of annual precipitation. The relative humidity in Liangshui is about 78%. The average annual evaporation is 805 mm, the total sunshine hours are 1848h, frost-free period is 100-120 days, the annual snow period is 130-150 days, frozen soil depth is about 2 m (Liu et al. 2014, Su et al. 2006). The forest types in this region mainly include five types of forest, namely Ulmus davidiana var. japonica—*Pinus koraiensis* forest (UPF), *Betula costata*—*Pinus koraiensis* forest (BPF), *Quercus mongolica*—*Pinus koraiensis* forest (QPF), *Fraxinus mandshurica*—*Pinus koraiensis* forest (FPF), *Tilia*-—*Pinus koraiensis* forest (TPF).

### Funding

Fundamental Research Funds for the Central Universities (2572015CA14); Construction of the plant checklist database in Heilongjiang province.

## Sampling methods

### Study extent

The dataset was collected between August 2016 and August 2017.

### Sampling description

A square plot approach was chosen to sample forest diversity. A total of five major forest types were selected, namely Ulmus davidiana var. japonica—*Pinus koraiensis* forest, *Betula costata*—*Pinus koraiensis* forest, *Quercus mongolica*—*Pinus koraiensis* forest, *Fraxinus mandshurica*—*Pinus koraiensis* forest, *Tilia*—*Pinus koraiensis* forest. Each forest type was set with 3 tree quadrats, 12 shrub quadrats with sampling from all the four corners and 12 herbaceous quadrats. The size of the tree quadrate was 625 m^2^ (25 × 25 m) which had four shrub plots (5 × 5 m) distributed on the four corners. Shrub plots 25 m^2^ which also had four herbaceous plots (1 × 1 m) distributed on the four corners. The plant species name were identified according to *The researches of liangshui natural* (Ma 1993), *Arbor flora of Heilongjiang Province* (Zhou 1986), *Flora plantarum herbacearum chinae boreali–orientalis* (Liou 1959), *Silva of Heilongjiang* (Zhou 1985), *Flora of China* (Wu et al. 2013), *Key of Plants of Northeastern China* (Fu 1995), *Flora Republicae Popularis Sinicae* (Editorial Committee of Flora of China of Chinese Academy Sciense 2004) and a plant checklist was obtained by survey. The number and height of each species were recorded for all plots, the crown of the trees was recorded, the coverage and total coverage of shrubs and herbs were also recorded.

### Quality control

The present dataset was updated to match the APGIV classification of angiosperm families (The Angiosperm Phylogeny Group 2016) and all species names were checked for validity (spelling and authorship) against online databases (http://tnrs.iplantcollaborative.org/index.html, http://ipni.org, http://plants.jstor.org, http://www.ville-ge.ch/musinfo/bd/cjb/africa/recherche.php).

### Step description

The dataset presented here was collected over a period of one year.

## Geographic coverage

### Description

Data was collected in five different forest types (Ulmus davidiana var. japonica—*Pinus koraiensis* forest, *Betula costata*—*Pinus koraiensis* forest, *Quercus mongolica*—*Pinus koraiensis* forest, *Fraxinus mandshurica*—*Pinus koraiensis* forest, *Tilia*—*Pinus koraiensis* forest) across the reserve as illustrated inTable 1 Table 2, Table 3, Table 4, Table 5, Table 6, Table 7, Table 8, Table 9, Table 10, Table 11 and Table 12 .

### Coordinates

 and Latitude; and Longitude.

## Taxonomic coverage

### Description

The dataset contains a total of 5007 tagged individuals representing 95 total taxa identified to species level belonging to 48 families and 78 genera (see Suppl. material 1).

## Temporal coverage

### Notes

Data range: 1 August 2016 - 1 August 2017.

## Usage rights

### Use license

Creative Commons Public Domain Waiver (CC-Zero)

## Data resources

### Data package title

List of all individuals established in all 5 forest types in Liangshui National Natural Reserve.

### Number of data sets

1

### Data set 1.

#### Data set name

raw_data_Lsnnr.xls - Download file

#### Number of columns

12

#### 

**Data set 1.** 

Column label	Column description
Forest type	Forest community types
Family_name	Plant family for the species follwing APG Ⅳ
Genus_name	Full scientific name of the genera in which the taxon is classified
Species_name	Name of the identified species
Plot number	Transect number
Quadrat size	Quadrat size in metres
Diameter at breast height (cm)	Diameter at breast height in centimetres
High (m)	Height of vegetations in metres
Canopy diameter (m)	Canopy diameter in metres
Number	The number of the plant
Total coverage	Coverage of all species in each quadrat
Coverage	Coverage of each species

## Supplementary Material

Supplementary material 1 List of all plants recorded in all 5 forest types in Liangshui National Natural Reserve. Data type: Excel file of List of all plants recorded in all 5 forest types in Liangshui National Natural Reserve
Brief description: This is the raw dataset of each plant individual recorded with their diameter at breast height, high, canopy diameter, as well as all shrubs and herbs recorded with their coverage, total coverage, number, height.
File: oo_175631.xls Wang Hongfeng & Dong Xueyun

## Figures and Tables

**Table 1.** Basic description of five forest types in Liangshui National Natural Reserve, where: UPF = *Ulmus davidiana* var. japonica—*Pinus koraiensis* forest; BPF = *Betula costata—Pinus koraiensis* forest; QPF = *Quercus mongolica—Pinus koraiensis* forest; FPF = *Fraxinus mandshurica—Pinus koraiensis* forest; TPF = *Tilia—Pinus koraiensis* forest.

**Forest types**	**Habitat**	**Coverage of** **shrub layer**	**Coverage of** **herb layer**	**Soil**
UPF	River bank or valley	40-50%	About 60%	Gravel soil, thick and humid, about 50-60 cm
BPF	Medium or gentle slope	50-60%	40-50%	Dark brown soil, fertility, about 25-30 cm
QPF	Sunny and steep slope	About 30%	30-40%	Gravel soil, barren and dry, 25-30 cm
FPF	River bank or valley	40-50%	About 60%	Gravel soil, thick and humid, about 50-60 cm
TPF	Medium or gentle slope	50-60%	40-50%	Dark brown soil, fertility, about 25-30 cm

**Table 2.** Geographical coordinates of quadrats of five forest types in Liangshui National Natural Reserve.

**Forest type**	**Plot number**	**North latitude**	**East longitude**
UPF	04-T	47°11′33.52"N	128°52′55.85"E
	11-T	47°11′2.41"N	128°53′59.93"E
	13-T	47°10′57.16"N	128°53′44.97"E
BPF	01-T	47°11′5.39"N	128°53′15.30″E
	12-T	47°11′0.43"N	128°53′45.52"E
	03-T	47°11′43.98"N	128°52′59.89”E
QPF	02-T	47°11′57.73"N	128°53′29.72"E
	14-T	47°8′32.22"N	128°54′3.04"E
	15-T	47°8′41.58"N	128°53′50.70"E
FPF	06-T	47°11′43.84"N	128°53′16.33"E
	07-T	47°10′56.62"N	128°53′32.46"E
	08-T	47°10′44.92"N	128°53′36.05"E
TPF	05-T	47°11′44.71"N	128°53′20.16"E
	09-T	47°10′48.91"N	128°53′39.81"E
	10-T	47°11′0.32"N	128°53′47.68"E

**Table 3.** The number of species in each family in Liangshui National Natural Reserve.

**Family**	**Species richness**
Rosaceae	11
Betulaceae	6
Umbelliferae	6
Pinaceae	5
Sapindaceae	5
Adoxaceae	3
Compositae	3
Cyperaceae	3
Fabaceae	3
Araliaceae	2
Asparagaceae	2
Dryopteridaceae	2
Gramineae	2
Oleaceae	2
Ranunculaceae	2
Rhamnaceae	2
Ruscaceae	2
Saxifragaceae	2
Tiliaceae	2
Ulmaceae	2
Urticaceae	2
Actinidiaceae	1
Amaranthaceae	1
Athyriaceae	1
Berberidaceae	1
Campanulaceae	1
Cannabaceae	1
Caprifoliaceae	1
Caryophyllaceae	1
Celastraceae	1
Cruciferae	1
Equisetaceae	1
Hydrangeaceae	1
Labiatae	1
Liliaceae	1
Malvaceae	1
Melanthiaceae	1
Oxalidaceae	1
Papaveraceae	1
Phrymaceae	1
Polemoniaceae	1
Primulaceae	1
Pteridaceae	1
Rubiaceae	1
Rutaceae	1
Salicaceae	1
Scrophulariaceae	1
Violaceae	1

**Table 4.** The number of individuals in each family of tree in Liangshui National Natural Reserve.

**Family**	**Number of individuals**
Pinaceae	535
Sapindaceae	462
Betulaceae	226
Tiliaceae	119
Ulmaceae	96
Fabaceae	43
Oleaceae	22
Salicaceae	20
Rosaceae	10
Rhamnaceae	6
Rutaceae	1

**Table 5.** The number of individuals in each family of shrub in Liangshui National Natural Reserve.

**Family**	**Number of individuals**
Araliaceae	177
Betulaceae	136
Adoxaceae	124
Caprifoliaceae	107
Rosaceae	77
Oleaceae	30
Berberidaceae	10
Sapindaceae	9
Hydrangeaceae	4
Celastraceae	3
Rhamnaceae	2
Actinidiaceae	1

**Table 6.** The number of individuals in each family of herb in Liangshui National Natural Reserve.

**Family**	**Number of individuals**
Oxalidaceae	445
Umbelliferae	332
Cyperaceae	305
Saxifragaceae	237
Rosaceae	231
Papaveraceae	194
Athyriaceae	171
Ruscaceae	114
Urticaceae	113
Adoxaceae	101
Gramineae	98
Compositae	55
Ranunculaceae	54
Amaranthaceae	53
Pteridaceae	40
Dryopteridaceae	38
Rubiaceae	37
Melanthiaceae	35
Violaceae	32
Labiatae	29
Asparagaceae	16
Fabaceae	10
Campanulaceae	7
Caryophyllaceae	7
Cruciferae	7
Liliaceae	6
Malvaceae	5
Cannabaceae	4
Polemoniaceae	3
Primulaceae	3
Equisetaceae	2
Phrymaceae	2
Scrophulariaceae	1

**Table 7.** The number of individuals in each species of tree in Liangshui National Natural Reserve. (Species names are checked by TPL)

**Species**	**Number of individuals**
Acer pictum subsp. mono Thunb.	270
*Picea koraiensis* Nakai	234
*Pinus koraiensis* Siebold & Zucc.	189
*Betula lenta* L.	162
*Acer ukurunduense* (Trautv. & C.A.Mey.) E.Murray	116
*Tilia amurensis* Rupr.	103
Ulmus davidiana var. japonica (Rehder) Nakai	80
*Acer tegmentosum* Maxim.	76
Picea jezoensis var. microsperma (Siebold & Zucc.) Carrière	72
*Betula platyphylla* Sukaczev	51
*Quercus mongolica* Fisch. ex Ledeb.	39
Pinus sylvestris var. mongolica Litv.	34
*Fraxinus mandshurica* Rupr.	22
*Populus davidiana* (Dode) C.K.Schneid.	20
*Tilia mandshurica* Rupr. & Maxim.	16
*Ulmus laciniata* (Trautv.) Mayr	16
*Betula dahurica* Pall.	9
*Larix gmelinii* (Rupr.) Kuzen.	6
*Rhamnus davurica* Pall.	6
*Amygdalus davidiana* (Carr.) Franch.	6
*Maackia amurensis* Rupr. et Maxim.	4
*Alnus hirsuta* (Spach) Rupr.	3
*Padus avium* L.	2
*Sorbus dacica* Borbás	2
*Alnus cremastogyne* Burkill	1
*Phellodendron amurense* Rupr.	1

**Table 8.** The number of individuals in each species of shrub in Liangshui National Natural Reserve.

**Species**	**Number of individuals**
*Eleutherococcus senticosus* (Rupr. & Maxim.) Maxim.	165
*Corylus mandshurica* (Maxim.) C.K.Schneid.	136
*Sambucus williamsii* Hance	124
*Lonicera japonica* Thunb.	107
*Sorbaria sorbifolia* (L.) A.Braun	51
Syringa reticulata subsp. amurensis (Rupr.) P.S.Green & M.C.Chang	30
*Cerasus verecunda* Koehne	21
Aralia elata var. glabrescens (Miq.) Seem.	12
*Berberis ferdinandi-coburgii* C.K.Schneid.	10
Acer tataricum subsp. ginnala (Maxim.) Wesm.	6
Acer pictum subsp. mono Thunb.	4
*Deutzia glabrata* Kom.	4
*Spiraea thunbergii* Siebold ex Blume	4
*Euonymus alatus* (Thunb.) Siebold	3
*Rhamnus davurica* Pall.	2
*Rosa davurica* Pall.	1

**Table 9.** The number of individuals in each species of herb in Liangshui National Natural Reserve.

**Species**	**Number of individuals**
*Oxalis griffithii* Edgew. & Hook. f.	445
*Carex leiorhyncha* C.A.Mey.	279
*Ostericum sieboldii* (Miq.) Nakai	278
*Chrysosplenium lectuscochleae* Kitag.	236
*Filipendula palmata* (Pall.) Maxim.	213
*Hylomecon japonica* (Thunb.) Prantl & Kündig	194
*Athyrium brevifrons* Nakai ex Kitag.	171
*Maianthemum bifolium* (L.) F.W.Schmidt	109
*Urtica angustifolia* Fisch. ex Hornem.	104
*Agrostis clavata* Trin.	97
*Adoxa moschatellina* L.	73
*Chenopodium album* L.	53
*Enemion raddeanum* Regel	52
*Sanicula rubriflora* F. Schmidt ex Maxim.	44
*Adiantum pedatum* L.	40
*Saussurea alatipes* Hemsl.	38
*Galium spurium* L.	37
*Paris verticillata* M.Bieb.	35
*Viola chaerophylloides* (Regel) W. Becker	32
*Meehania urticifolia* (Miq.) Makino	29
*Sambucus javanica* Blume	28
*Polystichum tripteron* (Kunze) C. Presl	27
*Carex breviculmis* R.Br.	23
*Parasenecio hastatus* (Linn.) H. Koyama	16
*Geum aleppicum* Jacq.	12
*Dryopteris crassirhizoma* Nakai	11
*Lathyrus davidii* Hance	10
*Polygonatum odoratum* (Mill.) Druce	10
*Urtica laetevirens* Maxim.	9
*Adenophora tetraphylla* (Thunb.) A.DC.	7
*Cardamine leucantha* (Tausch) O.E.Schulz	7
*Stellaria radians* L.	7
*Asparagus oligoclonos* Maxim.	6
*Lilium distichum* Nakai ex Kamib.	6
*Abutilon theophrasti* Medik.	5
*Agrimonia pilosa* Ledeb.	5
*Convallaria majalis* L.	5
*Humulus scandens* (Lour.) Merr.	4
*Carex siderosticta* Hance	3
*Heracleum hemsleyanum* Diels	3
*Ostericum grosseserratum* Maxim.	3
*Polemonium caeruleum* L.	3
*Trientalis europaea* (L.) U.Manns & Anderb.	3
*Aconitum volubile* Pall. ex Koelle	2
*Carum buriaticum* Turcz.	2
*Equisetum hyemale* L.	2
*Peucedanum praeruptorum* Dunn	2
Phryma leptostachya subsp. asiatica (Koidz.) Honda	2
*Artemisia stolonifera* Maxim.	1
*Chrysosplenium sinicum* Maxim.	1
*Fragaria orientalis* Losinsk.	1
*Miscanthus sacchariflorus* (Maxim.) Hack.	1
*Pseudolysimachion spurium* L.	1

**Table 10.** The characteristics of tree community of five forest types in Liangshui National Natural Reserve, where: UPF = Ulmus davidiana var. japonica—*Pinus koraiensis* forest; BPF = *Betula costata*—*Pinus koraiensis* forest; QPF = *Quercus mongolica*—*Pinus koraiensis* forest; FPF = *Fraxinus mandshurica*—*Pinus koraiensis* forest; TPF = *Tilia*—*Pinus koraiensis* forest; DBH = diameter at breast height; N = number.

**Families/Species**	**UPF**		**BPF**		**QPF**		**FPF**		**TPF**	
	N	DBH (cm)	N	DBH (cm)	N	DBH (cm)	N	DBH (cm)	N	DBH (cm)
** Betulaceae **										
*Alnus cremastogyne* Burkill	-	-	-	-	-	-	1	10.5	-	-
*Alnus hirsuta* (Spach) Rupr.	-	-	-	-	-	-	3	16.87	-	-
*Betula dahurica* Pall.	-	-	-	-	9	22.55	-	-	-	-
*Betula lenta* Ehrh.	-	-	112	18.56	28	22.96	4	13.8	18	10.14
*Betula platyphylla* Sukaczev	-	-	44	19.69	2	28	3	18.33	2	19.75
** Fabaceae **										
*Maackia amurensis* Rupr. et Maxim.	-	-	-	-	4	14.6	-	-	-	-
*Quercus mongolica* Fisch. ex Ledeb.	-	-	-	-	39	17.86	-	-	-	-
** Oleaceae **										
*Fraxinus mandshurica* Rupr.	1	33	-	-	1	2.8	5	16.44	15	29.23
** Pinaceae **										
*Larix gmelinii* (Rupr.) Kuzen.	-	-	6	11.87	-	-	-	-	-	-
Picea jezoensis var. microsperma (Siebold & Zucc.) Carrière	3	25.17	38	11.36	18	7.07	8	15.99	5	37.3
*Picea koraiensis* Nakai	32	14.46	112	9.86	55	7.47	17	15.76	18	8.58
*Pinus koraiensis* Siebold & Zucc.	21	51.45	64	23.19	60	12.79	13	49.59	31	46.92
Pinus sylvestris var. mongolica Litv.	-	-	12	5.75	22	1.77	-	-	-	-
** Rhamnaceae **										
*Rhamnus davurica* Pall.	-	-	6	5.2	-	-	-	-	-	-
** Rosaceae **										
*Amygdalus davidiana* (Carr.) Franch.	-	-	4	4	-	-	-	-	2	21
*Padus avium* L.	-	-	-	-	1	8	1	4.5	-	-
*Sorbus dacica* Borbás	-	-	-	-	-	-	-	-	2	5.5
** Rutaceae **										
*Phellodendron amurense* Rupr.	-	-	-	-	1	20	-	-	-	-
** Salicaceae **										
*Populus davidiana* (Dode) C.K.Schneid.	2	7.1	-	-	18	16.5	-	-	-	-
** Sapindaceae **										
Acer pictum subsp. mono Thunb.	29	7	47	5.75	60	8.16	57	6.03	77	5.42
*Acer tegmentosum* Maxim.	24	3.88	3	4.67	2	4.4	12	5.6	35	6.55
*Acer ukurunduense* (Trautv. & C.A.Mey.) E.Murray	19	6.27	18	4.74	53	12.39	17	3.09	9	3.53
** Tiliaceae **										
*Tilia amurensis* Rupr.	10	17.85	17	11.86	21	12.18	7	17.06	48	16.59
*Tilia mandshurica* Rupr. & Maxim.	-	-	-	-	2	14.5	1	3	13	10.23
** Ulmaceae **										
Ulmus davidiana var. japonica (Rehder) Nakai	45	19.35	1	25	13	37.52	12	6.86	9	4.38
*Ulmus laciniata* (Trautv.) Mayr	7	23.48	2	3.5	2	26	1	3	4	2.33

**Table 11.** The characteristics of shrub community of five forest types in Liangshui National Natural Reserve, where: UPF = Ulmus davidiana var. japonica—*Pinus koraiensis* forest; BPF = *Betula costata*—*Pinus koraiensis* forest; QPF = *Quercus mongolica*—*Pinus koraiensis* forest; FPF = *Fraxinus mandshurica*—*Pinus koraiensis* forest; TPF = *Tilia*—*Pinus koraiensis* forest; N = number; H = high.

**Families/Species**	**UPF**		**BPF**		**QPF**		**FPF**		**TPF**	
	N	H(m)	N	H(m)	N	H(m)	N	H(m)	N	H(m)
** Actinidiaceae **										
*Actinidia kolomikta* (Rupr. & Maxim.) Maxim.	-	-	-	-	1	1.5	-	-	-	-
** Adoxaceae **										
*Sambucus williamsii* Hance	24	0.99	12	1.33	6	1.23	36	1.19	46	0.91
** Araliaceae **										
Aralia elata var. glabrescens (Miq.) Seem.	-	-	-	-	10	0.93	-	-	2	2
*Eleutherococcus senticosus* (Rupr. & Maxim.) Maxim.	51	1.1	14	0.65	15	0.41	60	0.82	25	0.75
** Berberidaceae **										
*Berberis ferdinandi-coburgii* C.K.Schneid.	-	-	-	-	-	-	10	0.47	-	-
** Betulaceae **										
*Corylus mandshurica (Maxim.)* C.K.Schneid.	23	1.87	23	1.8	45	1.47	16	0.95	29	2.04
** Caprifoliaceae **										
*Lonicera japonica* Thunb.	24	0.9	18	0.83	40	1.38	20	0.93	5	1
** Celastraceae **										
*Euonymus alatus* (Thunb.) Siebold	-	-	2	1.5	-	-	-	-	1	3.5
** Hydrangeaceae **										
*Deutzia glabrata* Kom.	-	-	-	-	4	0.4	-	-	-	-
** Oleaceae **										
Syringa reticulata subsp. amurensis (Rupr.) P.S.Green & M.C.Chang	4	5	15	2.54	11	1	-	-	-	-
** Rhamnaceae **										
*Rhamnus davurica* Pall.	2	1.8	-	-	-	-	-	-	-	-
** Rosaceae **										
*Cerasus verecunda* Koehne	9	0.7	-	-	-	-	12	0.55	-	-
*Rosa davurica* Pall.	-	-	-	-	1	0.8	-	-	-	-
*Sorbaria sorbifolia* (L.) A.Braun	7	1.45	37	1.15	-	-	7	1.2	-	-
*Spiraea thunbergii* Siebold ex Blume	-	-	-	-	-	-	4	0.8	-	-
** Sapindaceae **										
Acer pictum subsp. mono Thunb.	-	-	4	1.75	-	-	-	-	-	-
Acer tataricum subsp. ginnala (Maxim.) Wesm.	-	-	-	-	5	1.2	-	-	-	-

**Table 12.** The characteristics of herb community of five forest types in Liangshui National Natural Reserve, where: UPF = *Ulmus davidiana* var. *japonica—Pinus koraiensis* forest; BPF = *Betula costata*—*Pinus koraiensis* forest; QPF = *Quercus mongolica—Pinus koraiensis* forest; FPF = *Fraxinus mandshurica*—*Pinus koraiensis* forest; TPF = *Tilia*—*Pinus koraiensis* forest; N = number=N; H = high.

**Families/Species**	UPF		BPF		QPF		FPF		TPF	
	N	H(m)	N	H(m)	N	H(m)	N	H(m)	N	H(m)
** Adoxaceae **										
*Adoxa moschatellina* L.	13	0.06	9	0.03	38	0.07	1	0.15	12	0.14
*Sambucus javanica* Blume	25	0.3	1	0.3	-	-	2	0.3	-	-
** Amaranthaceae **										
*Chenopodium album* L.	18	0.28	-	-	-	-	24	0.22	11	0.17
** Asparagaceae **										
*Polygonatum odoratum* (Mill.) Druce	-	-	10	0.33	-	-	-	-	-	-
*Asparagus oligoclonos* Maxim.	-	-	-	-	-	-	-	-	6	0.01
** Athyriaceae **										
*Athyrium brevifrons* Nakai ex Kitag.	10	0.46	82	0.23	14	0.25	51	0.34	14	0.32
** Campanulaceae **										
*Adenophora tetraphylla* (Thunb.) A.DC.	-	-	-	-	7	0.15	-	-	-	-
** Cannabaceae **										
*Humulus scandens* (Lour.) Merr.	3	1.2	-	-	1	0.2	-	-	-	-
** Caryophyllaceae **										
*Stellaria radians* L.	7	0.03	-	-	-	-	-	-	-	-
** Compositae **										
*Artemisia stolonifera* Maxim.	1	0.3	-	-	-	-	-	-	-	-
*Parasenecio hastatus* (Linn.) H. Koyama	9	0.45	7	0.23	-	-	-	-	-	-
*Saussurea alatipes* Hemsl.	30	0.45	5	0.3	-	-	-	-	3	0.6
** Cruciferae **										
*Cardamine leucantha* (Tausch) O.E.Schulz	3	0.3	-	-	-	-	4	0.2	-	-
** Cyperaceae **										
*Carex breviculmis* R.Br.	7	0.3	11	0.6	-	-	-	-	5	0.15
*Carex leiorhyncha* C.A.Mey.	10	0.35	28	0.3	140	0.29	74	0.38	27	0.2
*Carex siderosticta* Hance	-	-	-	-	-	-	3	0.1	-	-
** Dryopteridaceae **										
*Dryopteris crassirhizoma* Nakai	4	0.5	1	0.3	1	0.6	3	0.7	2	0.5
*Polystichum tripteron* (Kunze) C. Presl	-	-	18	0.25	-	-	1	0.15	8	0.1
** Equisetaceae **										
*Equisetum hyemale* L.	-	-	-	-	-	-	-	-	2	0.6
** Fabaceae **										
*Lathyrus davidii* Hance	-	-	-	-	10	0.16	-	-	-	-
** Gramineae **										
*Agrostis clavata* Trin.	53	0.28	44	0.28	-	-	-	-	-	-
*Miscanthus sacchariflorus* (Maxim.) Hack.	-	-	-	-	-	-	1	0.3	-	-
** Labiatae **										
*Meehania urticifolia* (Miq.) Makino	-	-	-	-	29	0.05	-	-	-	-
** Liliaceae **										
*Lilium distichum* Nakai ex Kamib.	-	-	-	-	3	0.28	3	0.2	-	-
** Malvaceae **										
*Abutilon theophrasti* Medik.	5	0.05	-	-	-	-	-	-	-	-
** Melanthiaceae **										
*Paris verticillata* M.Bieb.	-	-	2	0.15	33	0.1	-	-	-	-
** Oxalidaceae **										
*Oxalis griffithii* Edgew. & Hook. f.	62	0.06	51	0.04	46	0.06	176	0.08	110	0.04
** Papaveraceae **										
*Hylomecon japonica* (Thunb.) Prantl & Kündig	38	0.4	-	-	23	0.22	77	0.06	56	0.06
** Phrymaceae **										
Phryma leptostachya subsp. asiatica (Koidz.) Honda	2	0.6	-	-	-	-	-	-	-	-
** Polemoniaceae **										
*Polemonium caeruleum* L.	-	-	-	-	-	-	-	-	3	0.2
** Primulaceae **										
*Trientalis europaea* (L.) U.Manns & Anderb.	-	-	-	-	-	-	-	-	3	0.03
** Pteridaceae **										
*Adiantum pedatum* L.	-	-	-	-	-	-	40	0.25	-	-
** Ranunculaceae **										
*Aconitum volubile* Pall. ex Koelle	-	-	-	-	-	-	-	-	2	0.5
*Enemion raddeanum* Regel	5	0.15	11	0.23	3	0.1	33	0.26	-	-
** Rosaceae **										
*Agrimonia pilosa* Ledeb.	5	0.25	-	-	-	-	-	-	-	-
*Filipendula palmata* (Pall.) Maxim.	33	0.41	36	0.27	37	0.22	97	0.22	10	0.33
*Fragaria orientalis* Losinsk.	-	-	-	-	1	0.15	-	-	-	-
*Geum aleppicum* Jacq.	12	0.43	-	-	-	-	-	-	-	-
** Rubiaceae **										
*Galium spurium* L.	-	-	-	-	33	0.15	-	-	4	0.02
** Ruscaceae **										
*Maianthemum bifolium* (L.) F.W.Schmidt	5	0.07	89	0.05	-	-	4	0.04	11	0.05
*Convallaria majalis* L.	-	-	-	-	3	0.2	2	0.15	-	-
** Saxifragaceae **										
*Chrysosplenium lectus-cochleae* Kitag.	127	0.13	4	0.2	-	-	15	0.19	90	0.12
*Chrysosplenium sinicum* Maxim.	1	0.18	-	-	-	-	-	-	-	-
** Scrophulariaceae **										
*Pseudolysimachion spurium* L.	-	-	-	-	-	-	-	-	1	0.6
** Umbelliferae **										
*Carum buriaticum* Turcz.	-	-	2	0.45	-	-	-	-	-	-
*Heracleum hemsleyanum* Diels	-	-	-	-	-	-	3	0.3	-	-
*Ostericum grosseserratum* Maxim.	-	-	-	-	3	0.06	-	-	-	-
*Ostericum sieboldii* (Miq.) Nakai	38	0.09	63	0.09	30	0.08	35	0.08	112	0.07
*Peucedanum praeruptorum* Dunn	-	-	-	-	2	0.2	-	-	-	-
*Sanicula rubriflora* F. Schmidt ex Maxim.	7	0.18	-	-	1	1.2	36	0.18	-	-
** Urticaceae **										
*Urtica angustifolia* Fisch. ex Hornem.	20	0.4	-	-	17	0.25	24	0.3	43	0.39
*Urtica laetevirens* Maxim.	5	0.65	-	-	-	-	1	0.04	3	0.4
** Violaceae **										
*Viola chaerophylloides* (Regel) W. Becker	4	0.1	6	0.25	22	0.2	-	-	-	-
